# BrainTumNet: multi-task deep learning framework for brain tumor segmentation and classification using adaptive masked transformers

**DOI:** 10.3389/fonc.2025.1585891

**Published:** 2025-05-20

**Authors:** Cheng Lv, Xu-Jun Shu, Quan Liang, Jun Qiu, Zi-Cheng Xiong, Jing bo Ye, Shang bo Li, Cheng Qing Liu, Jing Zhen Niu, Sheng-Bo Chen, Hong Rao

**Affiliations:** ^1^ School of Mathematics and Computer Sciences, Nanchang University, Nanchang, Jiangxi, China; ^2^ Department of Neurosurgery, General Hospital of Eastern Theater Command, Nanjing, China; ^3^ Department of Neurosurgery, Affiliated Jinling Hospital, Medical School of Nanjing University, Nanjing, China; ^4^ Department of Radiology, Jinling Hospital, Nanjing, China; ^5^ Department of Critical Care Medicine, The Second People’s Hospital of Yibin, Yibin, Sichuan, China; ^6^ Department of Computer and Information Engineering, Henan University, Nanchang, China; ^7^ School of Software, Nanchang University, Nanchang, Jiangxi, China

**Keywords:** brain tumor diagnosis, deep learning, multi-task learning, medical image analysis, Convolutional Neural Networks

## Abstract

**Background and objective:**

Accurate diagnosis of brain tumors significantly impacts patient prognosis and treatment planning. Traditional diagnostic methods primarily rely on clinicians’ subjective interpretation of medical images, which is heavily dependent on physician experience and limited by time consumption, fatigue, and inconsistent diagnoses. Recently, deep learning technologies, particularly Convolutional Neural Networks (CNN), have achieved breakthrough advances in medical image analysis, offering a new paradigm for automated precise diagnosis. However, existing research largely focuses on single-task modeling, lacking comprehensive solutions that integrate tumor segmentation with classification diagnosis. This study aims to develop a multi-task deep learning model for precise brain tumor segmentation and type classification.

**Methods:**

The study included 485 pathologically confirmed cases, comprising T1-enhanced MRI sequence images of high-grade gliomas, metastatic tumors, and meningiomas. The dataset was proportionally divided into training (378 cases), testing (109 cases), and external validation (51 cases) sets. We designed and implemented BrainTumNet, a deep learning-based multi-task framework featuring an improved encoder-decoder architecture, adaptive masked Transformer, and multi-scale feature fusion strategy to simultaneously perform tumor region segmentation and pathological type classification. Five-fold cross-validation was employed for result verification.

**Results:**

In the test set evaluation, BrainTumNet achieved an Intersection over Union (IoU) of 0.921, Hausdorff Distance (HD) of 12.13, and Dice Similarity Coefficient (DSC) of 0.91 for tumor segmentation. For tumor classification, it attained a classification accuracy of 93.4% with an Area Under the ROC Curve (AUC) of 0.96. Performance remained stable on the external validation set, confirming the model’s generalization capability.

**Conclusion:**

The proposed BrainTumNet model achieves high-precision diagnosis of brain tumor segmentation and classification through a multi-task learning strategy. Experimental results demonstrate the model’s strong potential for clinical application, providing objective and reliable auxiliary information for preoperative assessment and treatment decision-making in brain tumor cases.

## 1 Introduction

Brain tumors are common and severe central nervous system diseases where early detection and accurate diagnosis are crucial for treatment planning and prognosis evaluation. The three most common types of brain tumors - gliomas, metastatic tumors, and meningiomas - present similar imaging characteristics. Traditional diagnostic procedures primarily rely on radiologists’ interpretation of Magnetic Resonance Imaging (MRI) data. However, this subjective assessment method has significant limitations in efficiency, consistency, and accuracy. With continuous advancement in medical imaging equipment and accumulation of clinical data, artificial intelligence-based diagnostic support systems have shown immense potential for application (1).Deep learning technologies, particularly Convolutional Neural Networks (CNN), have achieved breakthrough progress in medical image analysis. From the initial LeNet to the revolutionary AlexNet, and then to deeper architectures like VGGNet and ResNet, deep learning models have continuously improved in feature extraction and pattern recognition capabilities. In recent years, specialized networks for medical image segmentation, such as U-Net and V-Net, have further advanced medical image analysis. Notably, the successful application of Transformer architecture in computer vision has opened new research directions in medical image processing, with Vision Transformer (ViT) and Swin Transformer models demonstrating exceptional performance in various medical imaging tasks.

Current research in intelligent brain tumor diagnosis primarily follows two directions: improving segmentation accuracy, which is prerequisite for 3D image reconstruction, neural navigation, and 3D printing technologies; and enhancing classification accuracy, which forms the foundation of intelligent diagnostic assistance. In terms of segmentation, researchers have proposed various improvement strategies: Ahmed et al. (2) designed a segmentation network based on 3D U-Net, achieving a Dice Similarity Coefficient (DSC) above 0.85 through residual connections and depth-separable convolutions; Wang et al. proposed an attention-enhanced segmentation network (3), integrating multi-scale feature extraction and spatial attention mechanisms, achieving significant performance improvements on the BraTS dataset. Regarding classification, Akhil et al. (4) utilized an improved ResNet structure to extract discriminative information from ROI features, achieving 92% classification accuracy through multi-modal data fusion strategies; Li et al.’s Transformer-based classification framework demonstrated superior performance to traditional CNN models through effective capture of global contextual information via self-attention mechanisms. However, existing research mostly adopts independent or sequential approaches to handle segmentation and classification tasks. This separated approach has several disadvantages: 1) high model training costs, requiring separate models for different tumors and tasks, 2) low computational efficiency potentially leading to inconsistent expressions, and 3) high operational costs of multiple dispersed models unsuitable for clinical applications.

Addressing these issues, this study proposes a novel network architecture, BrainTumNet, achieving unified modeling of brain tumor segmentation and classification. The network innovatively designs a dual-path feature extraction module, integrating CNN’s local feature learning capabilities with adaptive masked Transformer’s global modeling advantages. Through a multi-scale feature fusion mechanism, it achieves information complementarity and collaborative optimization between segmentation and classification tasks, solving automatic segmentation and classification of different tumors through an end-to-end one-stop model.

The main innovations of this study include: (1) proposing a unified multi-task learning framework BrainTumNet; (2) designing a feature fusion mechanism and adaptive mask attention mechanism to effectively integrate spatial and semantic information; (3) achieving superior comprehensive performance compared to existing methods, providing a reliable diagnostic assistance tool for clinical practice. This research presents a new technical solution for the development of intelligent medical image analysis.

## 2 Materials and methods

### 2.1 Experimental preprocessing

#### 2.1.1 Data collection and selection

**Figure 1:**
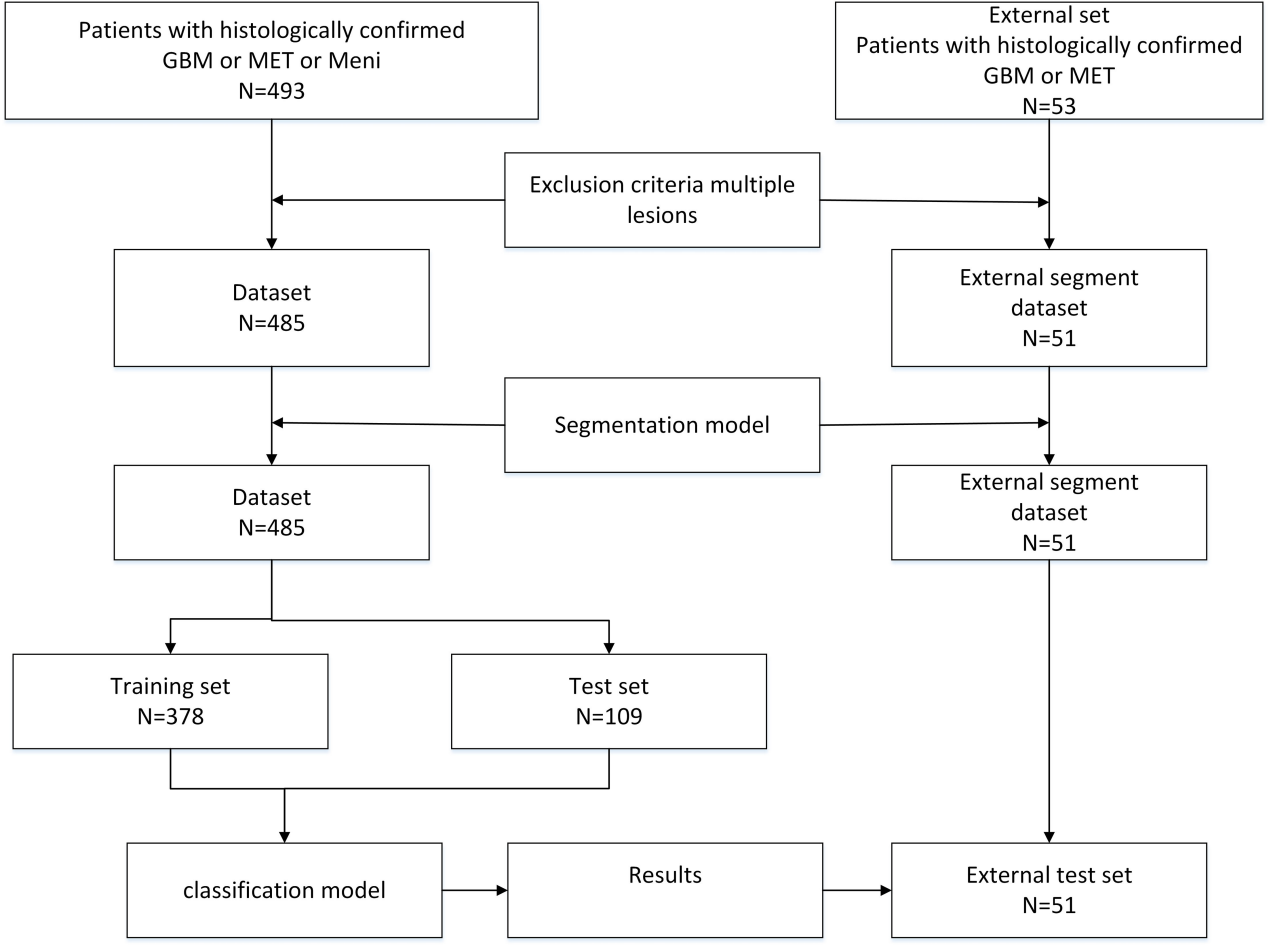
The flowchart of the braintumnet model, which sequentially enters the segmentation module and classification module to complete the process from segmentation to classification.

As shown in **Figure 1**, the study retrospectively utilized a dataset of 485 brain tumor cases, comprising 167 cases of gliomas, 156 cases of metastatic tumors, and 162 cases of meningiomas. Of these, 378 cases were allocated to the training set and 109 cases to the test set, with all data being screened by professional physicians. Each case consisted of CE-T1 magnetic resonance imaging, accompanied by pixel-level tumor segmentation annotations and pathological type labels. The brain tumor CE-T1 data were collected from Yibin Second People’s Hospital, Chinese PLA General Hospital, and Nanjing Jinling Hospital. The data acquisition was performed by professional physicians using three scanners: a 1.5T scanner (Erlangen, Siemens Espree, Germany) and a 3T scanner (GE) to obtain MRI images from all patients (6). Axial T1CE DICOM images were collected with a slice thickness of 1mm. The T1CE imaging parameters included a slice thickness of 1 mm, echo time of 3.02 ms, voxel dimensions of 0.997 × 0.997 × 1 mm³, matrix size of 512 × 512 × 176, field of view of 130 mm, repetition time of 1650 ms, and flip angle of 15°. Following the acquisition of the original DICOM format MRI data, standard data preprocessing procedures were implemented. This study was approved by the ethics committee of the Second People’s Hospital of Yibin City.

#### 2.1.2 Data preprocessing

The dataset was proportionally divided into training and testing sets for model training. Image data underwent initial preprocessing (5), where CE-T1 imaging modality data were normalized to the (0,1) interval. Data augmentation techniques, including random flipping and rotation, were applied. In the random rotation method, the rotation angle range was set from -30° to +30°, with random horizontal and vertical flips applied to images at a probability of 0.5. These image augmentation techniques enhance data diversity and enable the model to learn features from multiple perspectives. The 3D volumetric data were then sliced into 2D images and cropped to 256×256 dimensions, with 20 representative slices selected from each case, resulting in a total of 9,700 slices.

#### 2.1.3 Evaluation metrics

This study conducted comprehensive comparisons of various advanced image segmentation models for brain tumor segmentation tasks. The selected models included U-Net, nnU-Net, TransUNet, SwinUNet, and DeepLab, chosen for their exceptional performance and widespread application in image segmentation (7). The primary quantitative metrics for evaluating segmentation accuracy included the Dice Similarity Coefficient (DSC), Hausdorff Distance (HD), and Intersection over Union (IoU). These metrics comprehensively reflect model accuracy and robustness in segmentation tasks and are widely accepted in the field of image segmentation (8), providing an objective and standardized framework for model performance comparison.

In the experimental design for classification tasks, representative deep learning models including ResNet, DenseNet, and GoogleNet were selected for performance comparison. These models have been widely adopted for their efficiency and accuracy in image classification tasks. As shown in Equations 1–3, To comprehensively evaluate the classification performance of these models, the study employed statistical metrics including Accuracy (ACC), Sensitivity, Specificity, F1 Score, and Receiver Operating Characteristic (ROC) Curve. These metrics collectively form a multidimensional performance evaluation system, providing in-depth analysis of each model’s classification capabilities. Notably, the ROC curve offers a performance visualization across different threshold settings, while the F1 score provides a balanced measure of performance by considering both precision and recall.


(1) $$Accuracy=\frac{TP+TN}{TP+TN+FP+FN}$$

(2) $$Sensitivity=\frac{TP}{TP+FN}$$

(3) $$Specificity=\frac{TN}{TN+FP}$$

(4) $$HD=\max(max_{a\in A}\min_{b\in B}d(a,b),\max_{b\in B}\min_{a\in A}d(a,b))$$

In these metrics, TP (True Positive) represents the number of correctly predicted positive samples, TN (True Negative) represents the number of correctly predicted negative samples, FN (False Negative) represents the number of incorrectly predicted negative samples, and FP (False Positive) represents the number of incorrectly predicted positive samples.

#### 2.1.4 Training

The experiments employed 5-fold cross-validation on the training set. An Adam optimizer was utilized with an initial learning rate of 1e-4, which decayed according to a cosine strategy. The batch size was set to 16, and training continued for 250 epochs. The weights for segmentation and classification losses were set to 1.0 and 0.7, respectively (9). As shown in Equations 4–6, Dice Loss and DiceCELoss were selected as the loss functions. The final model performance was evaluated on the test set and compared with other methods.

The proposed model’s segmentation performance was compared with commonly used segmentation models including U-Net, nnU-Net, TransUNet, SwinUNet, and DeepLab. For tumor classification performance, comparisons were made with classifiers such as ResNet, GoogleNet, and DenseNet (10). Additionally, comparisons were conducted against two-stage (segmentation followed by classification) models. This experimental design and arrangement enables comprehensive evaluation of BrainTumNet’s segmentation and classification capabilities on glioma and metastatic tumor data, allowing fair comparison with other methods to validate the model’s effectiveness (11).

The study also analyzes the impact of key modules such as Transformer and Inception on model performance, providing a basis for model optimization. The rational selection of evaluation metrics and experimental procedures ensures the credibility and persuasiveness of the experimental results.


(5) $$DiceLoss=1-\frac{2\sum_{i}^{N}P_{i}g_{i}}{\sum_{i}^{N}P_{i}^{2}+\sum_{i}^{N}g_{i}^{2}}$$

(6) $$DiceCELoss=1-\frac{2\sum_{i}^{N}P_{i}g_{i}}{\sum_{i}^{N}P_{i}^{2}+\sum_{i}^{N}g_{i}^{2}}-\sum_{i}^{N}g_{i}\log(P_{i})$$

### 2.2 Model and architecture

**Figure 2:**
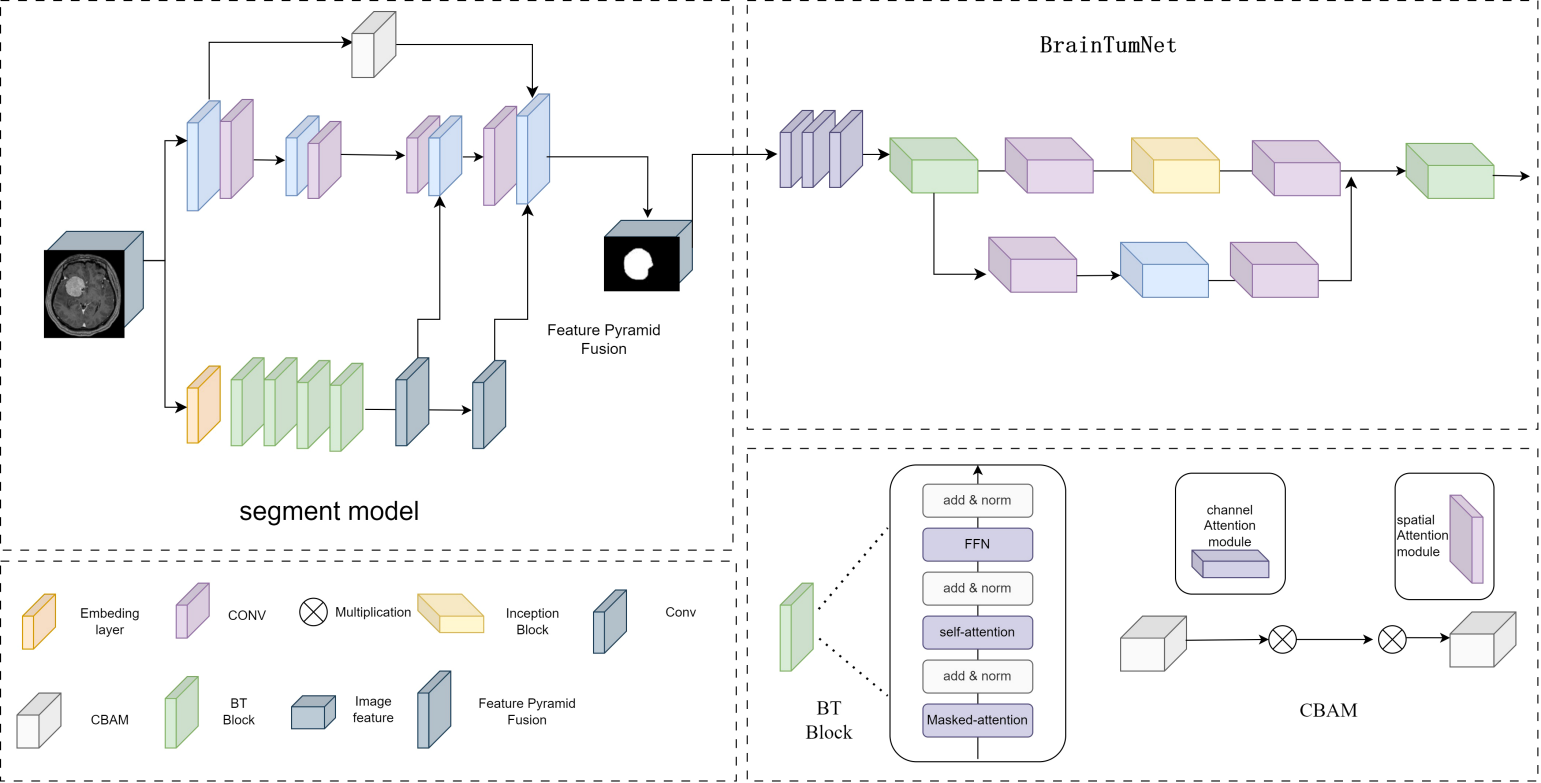
The model structure diagram of BrainTumNet, a brain tumor segmentation and classification model.

BrainTumNet is a dual-module network integrating segmentation and classification, designed for precise localization and diagnosis of brain tumors. As shown in **Figure 2**, the segmentation model adopts a convolutional encoder-decoder architecture. The encoder comprises multi-layer convolutions with downsampling structures, using different-sized convolutional layers for feature capture. The branch consists of multiple BT Blocks containing Masked attention, self-attention mechanisms, and MLP modules, effectively modeling long-range dependencies. The decoder employs a symmetric upsampling Pyramid multi-scale feature fusion strategy, with each layer including upsampling, skip-connection fusion CBAM modules, and BT Blocks for feature restoration (12). Skip-connect connections between encoder-decoder layers incorporate CBAM channel attention modules for enhanced feature fusion, effectively improving model segmentation accuracy (13). The module introduces adaptive masked transformer, which restricts attention to local regions within prediction masks, unlike traditional Transformer decoders that attend to all positions. The adaptive masked transformer divides input images into patches, transforms them through Patch Embedding, and uses a lightweight MLP network to predict patch importance scores, generating dynamic masks. This approach improves small object segmentation capability while reducing computational complexity.

The Adaptive Masked Transformer enhances model performance by dynamically adjusting the computational patterns of its attention mechanism. The core concept of this architecture enables the model to automatically determine the scope and intensity of attention computations based on input content, thereby optimizing computational efficiency while maintaining powerful feature extraction capabilities. In terms of structural design, the Adaptive Masked Transformer primarily consists of three key components: an Adaptive masked generator, a self-attention computation layer, and a feed-forward network. The dynamic mask generator analyzes the spatial distribution and semantic content of input features to generate unique soft masks for each attention head. These mask values are continuously distributed between 0 and 1, enabling precise control over the degree of attention computation participation at different positions. The self-attention computation layer incorporates both local window attention and global sparse attention modes, automatically selecting the most suitable computational approach for the current input features through learnable weight parameters. This Adaptive Masked Transformer architecture demonstrates significant advantages across multiple vision tasks. In terms of computational efficiency, benefiting from the dynamic masking mechanism, it reduces redundant computations by focusing on core regions.

The classification module T-InceptionNet extracts tumor region features from segmentation results. Its backbone comprises multiple T-Inception Blocks with parallel convolution modules (1x1, 3x3, 1x3, 3x1) suitable for capturing multi-scale feature patterns. The Inception-Transformer module employs various convolution sizes for feature capture and classification. The classification head consists of pooling, fully connected, and Dropout layers (14), supporting binary (glioma/metastatic) and multi-class output using Softmax activation.

The model adopts a two-stage cascade structure, performing segmentation followed by classification. BrainTumNet integrates Masked Transformer’s self-attention capabilities with CNN’s local perception abilities. The encoder excels at capturing long-range dependencies, while the decoder’s Skip-Transformer ensures feature propagation. The Inception module complements attention mechanisms through multi-scale feature extraction (15).

## 3 Results

The experimental study utilized an MRI dataset comprising 485 cases of gliomas (GBM), metastatic tumors (MET), and meningiomas, comprehensively evaluating BrainTumNet’s performance in tumor segmentation and classification tasks. The experiments compared several models including U-Net, nnU-Net, TransUNet, SwinUNet, and DeepLab, with comparative analyses demonstrating the superior performance of the proposed model.

In the segmentation experiments, the model was tested on all three tumor types. As shown in **Table 1**, for glioma segmentation, the U-Net model achieved an IoU score of 0.75 (16) and a Dice coefficient (DSC) of 0.752, demonstrating basic tumor region segmentation capability. nnU-Net showed improved performance with an IoU of 0.79 and DSC of 0.793. TransUNet performed better, achieving 0.80 and 0.815 for IoU and DSC respectively. SwinUNet also demonstrated excellent performance with scores of 0.874 and 0.88 (17). MedFormer achieved a DSC score of 89.5 and an IoU score of 0.89.BrainTumNet achieved a mean DSC of 0.902 on the test set, significantly outperforming U-Net (0.752), nnU-Net (0.793), and other classical segmentation models including TransUNet and SwinUNet. On the critical Hausdorff Distance (HD) metric, BrainTumNet achieved 12.13, substantially better than U-Net (32.21) and nnU-Net (22.53), validating its superior capability in precise tumor localization and boundary detail capture.

**Table 1:** The results of multiple segmentation models, including DSC, IoU, HD and other indicators.

Model	Glioma			Meningioma			Metastatic		
	Dsc↑	HD↓	IOU↑	DSC↑	HD↓	IOU↑	DSC↑	HD↓	IOU↑
Unet	75.2	32.21	0.75	76.1	31.61	0.76	73.8	29.13	0.73
Nnunet	79.3	22.53	0.79	79.1	21.8	0.81	79.1	22.6	0.78
Transunet	81.5	18.25	0.80	81.3	18.3	0.81	82.1	18.32	0.82
Deeplab	84.5	17.61	0.83	82.6	17.84	0.83	84.6	17.49	0.85
swinunet	87.4	15.16	0.88	87.6	15.73	0.87	88.3	14.47	0.88
Medformer	89.5	13.24	0.89	90.2	13.58	0.88	89.8	12.69	0.88
BrainTumNet	90.2	12.13	0.91	91.4	12.73	0.92	90.1	11.78	0.91

**Figure 3:**
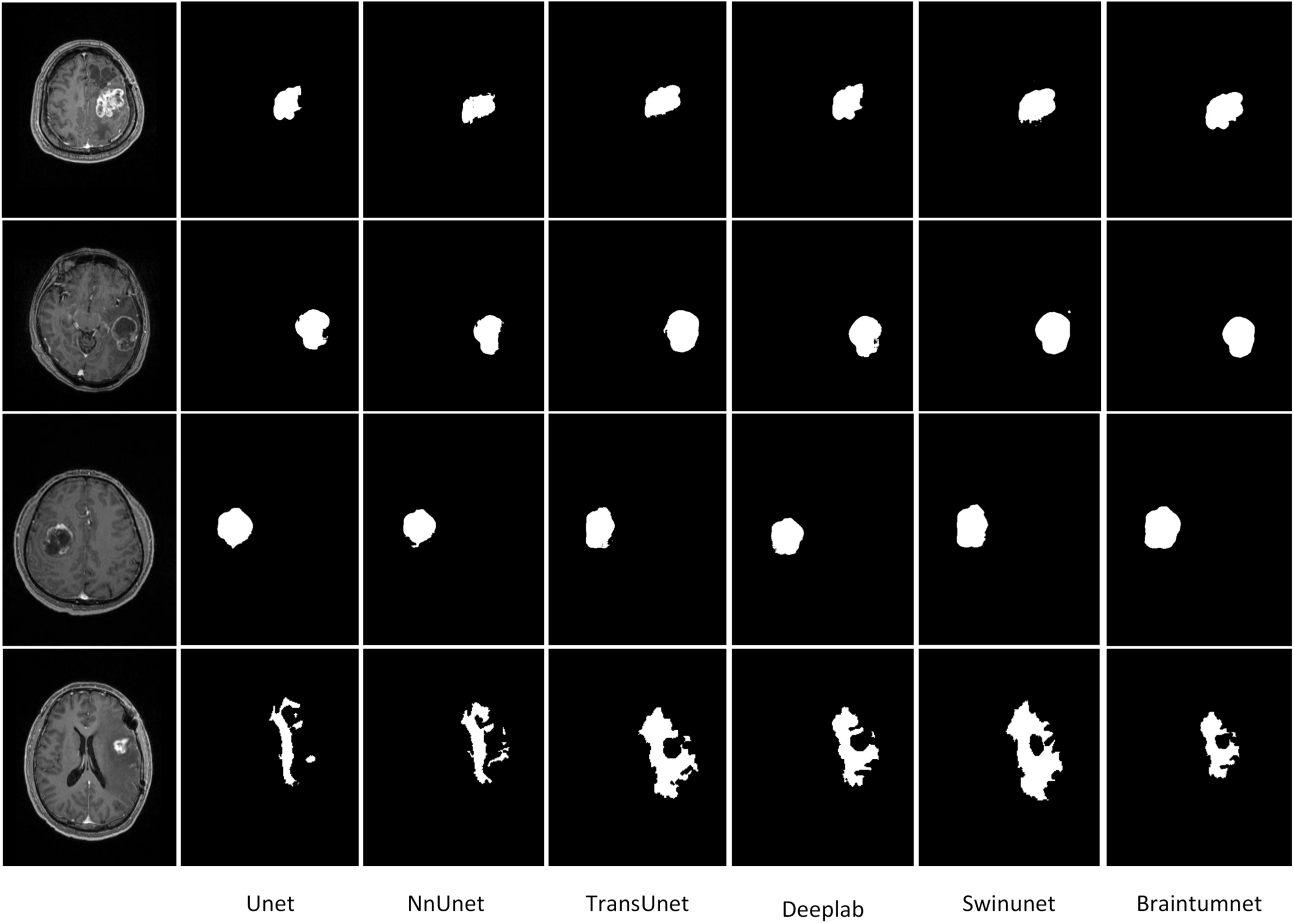
The segmentation results of brain tumors include the tumor areas of gliomas, metastases, and meningiomas, as well as the edematous parts.

For meningioma segmentation, As shown in **Figure 3**, U-Net achieved an IoU score of 0.76 and DSC of 0.761. nnU-Net demonstrated better performance with IoU and DSC scores of 0.81 and 0.791 respectively. TransUNet achieved an IoU of 0.81 and DSC of 0.813. SwinUNet’s performance approached that of BrainTumNet, achieving an IoU of 0.87 and DSC of 0.876. MedFormer achieved a DSC score of 90.2 and an IoU score of 0.88.BrainTumNet achieved an IoU of 0.92 and DSC of 0.914 in this task, further demonstrating its excellence in medical image segmentation.

**Figure 4:**
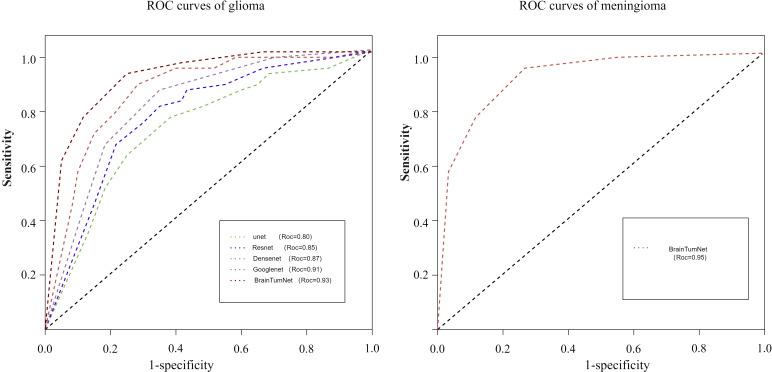
Roc curves of glioma and metastatic tumors.

Following segmentation and image preprocessing, the model was validated on the test set using five-fold cross-validation, evaluating accuracy for tumor, edema, and whole regions across three tumor types (18). As shown in **Table 2**, the tumor region achieved five-fold accuracies of 91%, 94%, 93%, 92%, and 93%, with a mean accuracy of 92.6%. The whole region achieved accuracies of 89%, 83%, 91%, 85%, and 87%, averaging 87.2%. For the edema region, the model achieved five-fold accuracies of 87%, 83%, 86%, 85%, and 85%, with a mean accuracy of 0.85. The model demonstrated highest recognition accuracy in the tumor region, while the edema region also reflected tumor type characteristics (19). As shown in **Figure 4**, BrainTumNet achieved a ROC score of 0.93.

**Table 2:** Accuracy table of various types of slices.

Model	Fold1	Fold2	Fold3	Fold4	Fold5	Average
Core	0.91	0.94	0.93	0.92	0.93	0.926
Overall parts	0.89	0.83	0.91	0.85	0.87	0.872
Edema	0.87	0.83	0.86	0.85	0.85	0.852

**Figure 5:**
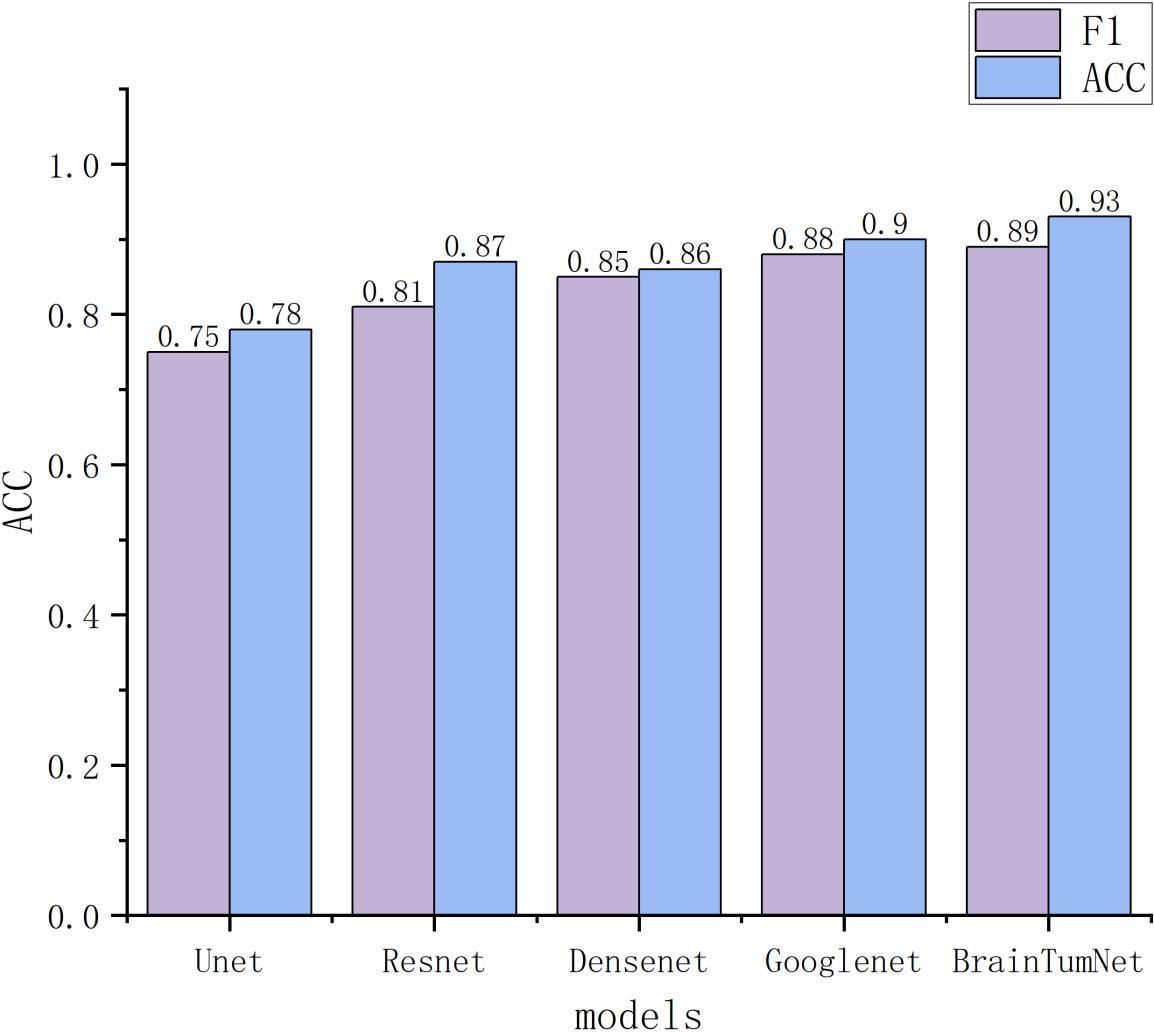
Classification accuracy and F1 score results of multiple models.

For classification tasks, as presented in **Table 3**, U-Net achieved 0.78 accuracy, ResNet reached 0.87, DenseNet performed better at 0.88, and GoogleNet achieved 0.90. BrainTumNet achieved a classification accuracy of 0.93 (20), surpassing standalone ResNet (0.87) and DenseNet (0.88) classifiers by 2–3 percentage points. As shown in **Figure 5**, the model achieved an F1 score of 0.89 (21), outperforming other models and demonstrating the significant advantages of incorporating auxiliary classification branches and pre-trained encoders. **Figure 4** displays BrainTumNet’s ROC curves for different tumor types, achieving an AUC value of 0.964.

**Table 3:** Classification accuracy of three types of brain tumors: gliomas, metastases, and meningiomas.

Model	Glioma			Meningioma			Metastatic		
	Sen	Spe	ACC	Sen	Spe	ACC	Sen	Spe	ACC
Unet	0.77	0.79	0.78	0.78	0.81	0.79	0.76	0.78	0.77
Resnet	0.85	0.89	0.87	0.85	0.87	0.86	0.83	0.85	0.84
Densenet	0.87	0.90	0.88	0.86	0.92	0.89	0.85	0.89	0.87
Googlenet	0.91	0.92	0.90	0.89	0.93	0.91	0.87	0.91	0.89
BrainTumNet	0.92	0.95	0.93	0.93	0.95	0.94	0.90	0.94	0.92

Further comparison with traditional two-stage segmentation-then-classification approaches showed that, under identical backbone networks and training configurations, the end-to-end multi-task model improved DSC by 2.1% in segmentation and classification accuracy by 2.7%, demonstrating the effectiveness and efficiency of multi-task learning compared to two-stage methods.

The external dataset is the MRI data set of 51 cases of glioma, metastatic tumor and meningioma, as shown in **Table 4**, the BrainTumNet demonstrates robust performance on external datasets, achieving an IoU of 91%, DSC of 92.1%, and HD of 11.47. These three segmentation metrics indicate stable performance. Furthermore, on the BraTS 2019 public dataset, the model achieves an IoU of 92% and DSC of 91.2%, surpassing the corresponding metrics of other segmentation methods, highlighting its superior segmentation capability.

**Table 4:** Image segmentation result table of public data set and external data set, including DSC, IoU, HD and other indicators.

Model	2019 BraTS			External dataset		
	Dsc↑	HD↓	IOU↑	DSC↑	HD↓	IOU↑
Unet	74.4	31.19	0.74	75.5	29.78	0.77
Nnunet	78.5	23.27	0.77	79.3	21.8	0.78
Transunet	80.8	19.48	0.81	82.3	17.2	0.81
Deeplab	83.9	19.27	0.84	84.6	17.84	0.83
swinunet	87.9	14.79	0.86	88.6	14.25	0.88
Medformer	89.7	12.12	0.88	90.2	12.12	0.89
BrainTumNet	91.2	11.25	0.92	92.1	11.47	0.91

In the evaluation of classification tasks on the External dataset, the performance metrics of various models are shown in **Table 5**. The results reveal significant differences among models with different architectures in terms of accuracy and area under the curve (AUC). Specifically, the UNet model achieved an accuracy of 0.77 and an AUC of 0.816, indicating relatively limited classification performance on this dataset. In contrast, the ResNet model improved accuracy to 0.81 and reached an AUC of 0.841, demonstrating enhanced classification ability. The DenseNet model further optimized performance, with an accuracy of 0.85 and an AUC of 0.918, highlighting its significant advantages in feature extraction and classification precision. The GoogLeNet model further enhanced classification performance with an accuracy of 0.89 and an AUC of 0.937, showcasing its robust feature learning capability. the BrainTumNet model stood out with an accuracy of 0.92 and an AUC of 0.964, achieving the best classification results.

**Table 5:** Accuracy of brain tumor classification in external data sets, including sensitivity, specificity, accuracy, AUC index.

Model	External dataset			
	Sen	Spe	ACC	AUC
Unet	0.76	0.78	0.77	0.816
Resnet	0.79	0.83	0.81	0.841
Densenet	0.84	0.86	0.85	0.918
Googlenet	0.87	0.91	0.89	0.937
BrainTumNet	0.90	0.94	0.92	0.964

**Figure 6:**
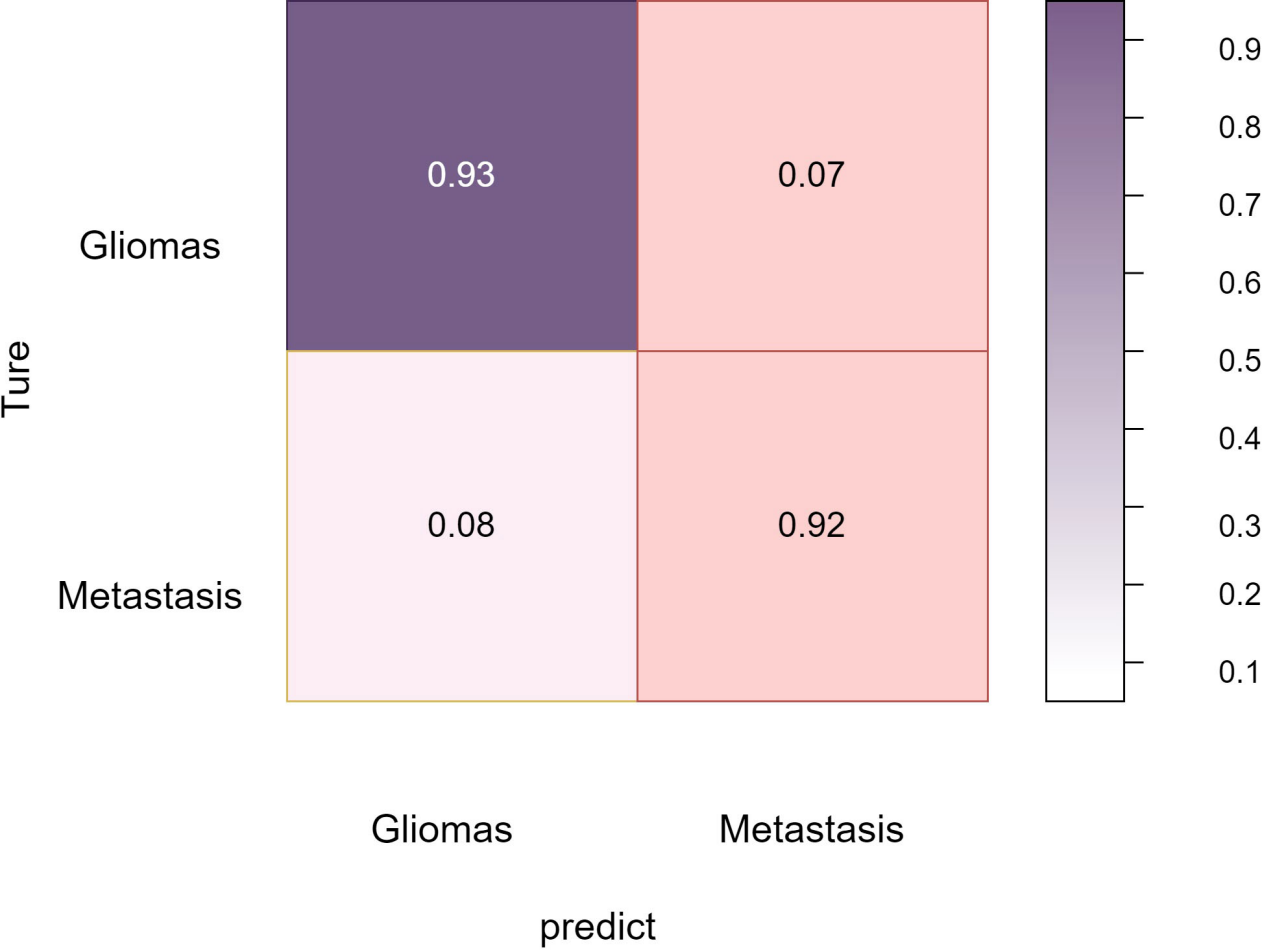
Glioma and metastatic tumor accuracy heatmap.

Overall, these comprehensive experimental results validate BrainTumNet’s exceptional performance in both brain tumor segmentation and classification tasks, demonstrating its significant potential for improving clinical diagnosis quality and efficiency (22). The model’s innovative design facilitates efficient extraction of visual and semantic features from medical images, enabling mutual enhancement between segmentation and classification tasks, thus improving diagnostic accuracy. The **Figure 6** reflects the classification accuracy of the two types of tumors: gliomas and metastatic tumors.

**Figure 7:**
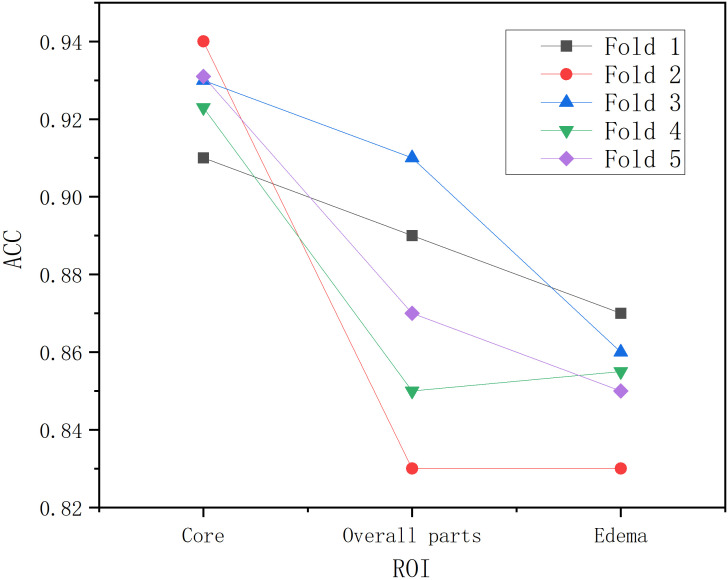
Accuracy chart of tumor, edema, and overall partial classification.

As shown in **Figure 7**, in the three-part image classification, BrainTumNet achieved higher classification accuracy in tumor regions compared to whole regions, with edema regions showing the lowest accuracy. The model’s excellent performance in both segmentation and classification tasks demonstrates its potential for improving brain tumor diagnostic accuracy and efficiency, providing an effective intelligent diagnostic tool for clinical practice.

## 4 Conclusion

This paper presents an innovative multi-task BrainTumNet model for simultaneous precise brain tumor segmentation and accurate classification. The model integrates advanced designs including pre-trained encoders and adaptive masked Transformer modules, enabling efficient extraction of visual and semantic features from medical images. BrainTumNet achieves mutual enhancement between segmentation and classification tasks, significantly improving overall performance.

We comprehensively evaluated the model on a dataset containing 485 cases of gliomas and metastatic tumors. In segmentation tasks, BrainTumNet achieved an average Dice similarity coefficient of 0.91 and Hausdorff distance of 12.3, both surpassing classical segmentation models like U-Net and nnU-Net, validating its excellence in precise tumor localization and boundary detail capture. For classification tasks, the model achieved 93.4% classification accuracy, 0.912 F1 score, and 0.964 AUC-ROC, significantly outperforming ResNet and DenseNet-based classifiers (23). The introduction of auxiliary classification branches and pre-trained encoders played crucial roles in improving classification performance.

Compared to traditional two-stage segmentation-then-classification workflows, BrainTumNet achieves functional unity, demonstrating the effectiveness of the multi-task learning paradigm. Our work demonstrates BrainTumNet’s immense potential in improving brain tumor diagnostic quality and efficiency.

## 5 Discussion

BrainTumNet’s key innovation lies in its efficient dual-branch structure, specifically designed for simultaneous segmentation and classification tasks. The segmentation branch employs convolutional blocks and adaptive masked transformers for feature recognition, precisely reconstructing tumor boundaries and locations (24). The classification branch utilizes multi-scale inception convolution modules combined with fully connected layers for tumor type prediction.

The adaptive masked Transformer’s core strength lies in the synergy between its mask generation mechanism and self-attention mechanism. A lightweight mask generation network dynamically produces spatial attention masks, adaptively adjusting pixel-wise or regional importance weights based on input image content (25). The mask generation network is jointly optimized using low-level and high-level semantic features (26), ensuring both local detail capture and global semantic information representation. Compared to other transformer-based segmentation models, TransUNet achieves global context modeling by incorporating Vision Transformer, yet its standard self-attention mechanism presents two critical limitations: it requires dense attention computations across all pixel positions (27), resulting in excessive computational complexity when processing high-resolution medical images; it employs a fixed attention pattern (28), lacking the ability to adaptively focus on key anatomical structures or lesion regions based on image content.

Although Swin-UNet reduces computational complexity by introducing hierarchical window-based attention mechanisms, its fixed window partitioning scheme lacks flexibility when handling multi-scale targets commonly found in medical images. Moreover, it requires complex window-shifting operations to facilitate cross-window information exchange. In contrast, the Adaptive Masked Transformer innovatively introduces a learnable dynamic masking mechanism that automatically generates attention weight distribution maps based on input image features (29). This enables the model to intelligently concentrate computational resources on diagnostically valuable key regions while significantly reducing computational overhead in irrelevant background areas. This content-aware sparse attention mechanism not only reduces computational complexity but also achieves faster inference speeds through efficient sparse matrix operations, allowing the architecture to process large-scale data such as high-precision medical images efficiently while maintaining high accuracy.

While the adaptive masked Transformer demonstrates significant advantages in image segmentation tasks (30), particularly in dynamic global context modeling and key region focusing, its computational complexity increases when processing high-resolution images or long sequence data (31). With the rapid advancement of Transformer technology in medical image analysis (32), its robust feature modeling capabilities have established a new research paradigm for multimodal medical data analysis. However, adaptive masked transformer models demonstrate limitations in processing multimodal data, as variations in resolution and contrast across different modalities increase the complexity of mask generation (33). Future research should focus on developing modality-adaptive dynamic masking strategies and constructing multi-branch mask generation networks (34), where each branch specifically processes feature distributions of distinct modalities, while optimizing for practical applications, addressing mask mechanism design and training stability challenges, maintaining performance, and reducing model sensitivity to hyperparameters.

## Data Availability

The original contributions presented in the study are included in the article/supplementary material. Further inquiries can be directed to the corresponding authors.
